# Among the Colleges

**Published:** 1898-08

**Authors:** 


### AMONG THE COLLEGES.

### KANSAS CITY VETERINARY COLLEGE.

THIS college was chartered in 1891, and while it started in a humble way as a private institution, its curriculum has been gradually broadened, until now it covers nearly, if not entirely, the full field of veterinary science. While its classes have been small their quality has continued to grow better, owing to an increased higher standard of matriculation examinations. During the college year of '97 and '98 some twenty-three students were in attendance. All of the freshmen class last year presented credentials of one or more terms of training in high schools. This preliminary school training is reported to have been most manifest in the excellent work done by the students and high grades obtained in final examinations.

The seventh annual commencement exercises of this collge were held at the Midland Hotel, Kansas City, Missouri, on Thursday, March 31st at 8.30 P.M. The degree of D.V.S. was conferred upon the following students: William N. D. Bird, John D. Cooper, Charles H. Davies, M.D., W. W. Johnston, M.D., and James Otterman, M.D. At the banquet Dr. C. J. Sihler presented the diplomas, the class response being delivered by Dr. W. W. Johnston. Dr. S. L. Brooking acted as toast-master; the following toasts were responded to: "Our Faculty," Dr. S. Stewart; "Medical and Veterinary Profession," Dr. I. J. Wolf; "The Veterinary Microbe," Dr. O. W. Krueger; "The Medical Man as a Fighter," Dr. Leon Rosenwald; "The Veterinarian's Place in Society," Dr. Geo. A. Johnson.

The college hospital affords a large clinic, where the students receive practical instruction in diagnosis, prognosis, and treatment. The location of the college in one of the great Western packing centres has afforded special opportunity for studies in meat-inspection, and it is to be noted that of the five graduates of March 30, 1898, the college calendar indicates that they are all employed by the Bureau of Animal Industry as meat-inspectors. The school seems to be specially favored in this direction by location and the fact that a large number of the instructors are engaged in this special work.

The school maintains a three-year graded course, and announces its compliance with the spirit as well as the letter of the agreement of those composing the Association of Veterinary Faculties establishing a minimum standard of requirements for all veterinary colleges, and the requirements for candidates for membership in the United States Veterinary Medical Association.

Of the forty-five graduates of the college, eleven are now empployed in the Bureau of Animal Industry. The following changes in the governing board and staff of teachers have been made for the ensuing year:

*Changes in Faculty.* E. J. Lutz, M.D., has severed his connection as Director of Histological and Pathological Laboratories. Richard J. Blanch, D.V.M., succeeds Sidney L. Hunter, D.V.S., as instructor in materia medica and therapeutics. O. W. Krueger, M.D., will teach histology, and J. C. Milnes, V.S., canine pathology. Charles H. Davies, M.D., D.V.S., will teach comparative anatomy in place of Frederick W. Hopkins, V.S. W. Ross Cooper will lecture on breeds and breeding, in place of U. B. McCurdy, D.V.S. Thomas W. Carnachan, M.R. C.V.S., will instruct in physical diagnosis and examination for soundness, succeeding W. A. Heck, D.V.M. Don W. Patten, D.V.M., as demonstrator of anatomy, succeeds Leslie J. Allen, V.S.

The following text-books have been added: *Obstetrics*, Dalrymple; *Foods and Feeding*, Henry; and as reference-books: *Parasites*, *Reports of Bureau of Animal Industry; Lameness and Shoeing*, Hunting; Adams' translation of *Lungwitz; Dictionary*, Duane.

### CHANGES IN INDIANA VETERINARY COLLEGE.

*Officers*—S. E. Crosse, A.M., M.D., succeeds as President E. H. Pritchard, V.S. Dr. Crosse is also Dean of the Faculty. In the Board of Trustees Thos. Gaddes, M.D., V.S., succeeds T. L. Armstrong, D.V.S., J. A. Mullan being added to the Trustees. In the teaching staff W. P. Garshwiler, M.D., Professor of Physiology, fills the place of John F. Geis, M.D.; the latter will fill the chair of Histology and Microscopic Pathology, formerly taught by E. H. Pritchard, V.S. John F. Roe, D.V.S., will teach Cattle Pathology and Contagious Diseases; F. A. Mueller, Ph.G., V.S., becomes Professor of Materia Medica and Therapeutics in place of A. J. Mullan, Ph.G., M.D. Dr. Mueller was formerly Professor of Pharmacy and Pharmaceutical Chemistry. E. H. Pritchard, V. S., resigned, was also Professor of Meat- and Milk-inspection and Contagious Diseases. The chairs of Pharmacy and Pharmaceutical Chemistry and of Meat- and Milk-inspection, and Demonstrator of Anatomy are provided for in this year's work by special lectures. As lecturers P. O. O'Rear, D.V.S., will teach Veterinary Dentistry in place of John F. Roe, D.V.S., and lectures on meat- and milk-inspection will be given by John F. Roe, D.V.S. Lectures on Pharmacy and Pharmaceutical Chemistry by F. A. Mueller, Ph.G., V.S. F. C. Becker, M.D., lecturer on Bacteriology, has severed his connection, and this course is placed under the lectures of Reginald W. Garstang, M.D., who formerly lectured on general pathology and morbid anatomy.

As a text-book *Meat-inspection*, by Walley, has been added.

### CHANGES IN McKILLIP VETERINARY COLLEGE.

*Faculty and Officers*—John B. Boomer, M.D.V., has been installed as Instructor of Veterinary Pharmacy.

Associated with clinical work connected with the college there have been added I. D. Rawlings, M.S., M.D., as Consulting Bacteriologist, and Leo. Thurlimann, M.S., Analytical Chemist. Several new text- and reference-books have been added to the list of books recommended.

### AMERICAN VETERINARY COLLEGE.

*Regents—Ex-officio* Frank S. Black, B.A., Governor, succeeds Levi P. Morton; Timothy L. Woodruff, M.A., Lieutenant-Governor, succeeds Charles B. Saxton, LL.D. Additional election by the Legislature—Chester S. Lord, M.A., Brooklyn, N. Y. Added to the Director of Departments —James Russell Parsons, J.M.A., Examination Department, and F. H. Merrill, Ph.D., State Museum.

*Governing Faculty*—H. H. Rawlings, B.S., succeeds L. H. Friedberg, Ph.D., as Professor of Chemistry and Toxicology.

*Adjunct Faculty*—Olof Schwarzkopf, V.M., becomes Professor of Surgical Pathology and Practical Microscopy; H. B. Ferguson, Ph.G., Phar. D., succeeds H. H. Rusby, M.D., as Professor of Botany. George C. Beckett, D.V.S., M.R.C., V.S., as Lecturer on Bacteriology; also H. Neher, D.V.S., as Clinical Professor of Veterinary Medicine and External Forms of the Horse, and Allen S. Heath, M.D., V.S., as Professor of Hygiene, Breeding Zootechny, have severed their connection with the teaching staff. R. W. Ellis, D.V.S., has been added as Lecturer on Obstetrics.

In the American Veterinary Hospital in connection with this college, E. F. Sanford, D.V.S., succeeds Roger Twombly as Assistant House Surgeon. H. Neher, D.V.S., has been added as one of the Visiting and Clinic Veterinarians.

*Text-books and Books of Reference—Dog Practice*, by Alexander Glass, V.S., has been added in place of that by J. Woodroffe Hill. Chemistry, have added Attfield's *General and Pharmaceutical Chemistry*, also Robert Friedberg's. *Surgical Pathology*, have added Gottheil's (in preparation). *Exterior of the Horse*, have added Percival's. Botany: Gray's *Lessons* in place of Rushby and Jeliffe's *Essentials*.

### VETERINARY DEPARTMENT IOWA STATE COLLEGE OF AGRICULTURE AND MECHANICAL ARTS.

THIS institution is located a mile and a half from the town of Ames, on a farm of nine hundred acres, the buildings being located in a beautiful park embracing more than a hundred acres. A motor line furnishes ready communication between the college buildings and the railway station in town. This college gives a three-years' course of six terms of seventeen weeks each. It is endowed, and thus is rendered independent of the fees. A thorough course of instruction has been adopted, embracing the most approved methods of theoretical and practical teaching.

Changes have been made for 1898 as follows: On Board of Trustees, Hon. W. J. Dixon, Sac City, succeeds Hon. H. C. Wheeler, Odebolt. Faculty—J. E. Bingham, Lecturer on Lameness and Principles of Shoeing, succeeds F. B. McCall, D.V.M.; C. F. Curtis, M.S.A., Professor of Principles of Heredity and Animal Nutrition, succeeds Hon. James Wilson; M. F. Patterson, M.D., succeeds A. R. Amos, M.D., Lecturer on Ophthalmology; W. B. Lincoln, D.V.M., House Surgeon and Demonstrator of Anatomy, succeeds L. L. Lewis, D.V.M.

The department library numbers over 200 volumes, selected with special reference to the instruction given in veterinary science. The general college library numbers about 9300 carefully selected volumes.

### WESTERN VETERINARY COLLEGE.

The Western Veterinary College, located at Kansas City, Mo., opened its doors in the fall of 1897, and graduated its first class of students, numbering five, in the spring of this year (1898).

Drs. J. H. Wattles and son, the senior being formerly connected with the Kansas City Veterinary College, are the prime movers in the new institution.

The announced Faculty for 1898–99 is composed of some eleven veterinarians and two practitioners of human medicine. In its opening announcement it says: This is a two-year school, and as such has no favors to ask and no apologies to make. In its preliminary announcement of 1897 it promised to be a three-years school and to in every way adhere to the requirements demanded by the U. S. V. M. A. and the Association of Faculties. It promises a rigid adherence to its roster of instruction by the entire-named Faculty.

The degree of doctor of veterinary science is conferred, and the lectures and examinations cover six months, from October 1st to March 31st.

A "Stockman's Course" is inaugurated, intended to partially educate men who purpose following stock-raising or dairy interests. This covers but one session.

A common-school education covers the entrance requirements. College fees are $75 per year, except to graduates of other colleges, who are required to pay but $50 per year.

Western veterinary colleges report an exceptionally large number of applicants for catalogues, and prospective students.

